# A New Vacuum Pressure Infiltration CFRP Method and Preparation Experimental Study of Composite

**DOI:** 10.3390/polym12020419

**Published:** 2020-02-12

**Authors:** Yuqin Ma, Jie Wang, Yatao Zhao, Xinliang Wei, Luyan Ju, Yi Chen

**Affiliations:** 1 School of Mechano-Electronic Engineering, Xidian University, Xi’an 710071, China; imustwj@163.com (J.W.); xidianyatao@163.com (Y.Z.); xdchenyi@163.com (Y.C.); 2 Army aviation institute, Beijing 101123, China; weixinliang1982@126.com; 3 Mechanical Engineering College, Xi’an Shiyou University, Xi’an 710065, China

**Keywords:** vacuum infiltration hot pressing, 2D-CFRP, preparation method, experimental system design

## Abstract

In order to prepare a carbon-fiber-reinforced polymer composite (CFRP) with ideal microstructure and properties, a new vacuum pressure infiltration CFRP method is proposed based on an analysis of existing CFRP preparation process methods. Research on composite material preparation systems was carried out by using this new method principle. The system mainly includes a fiber pre-forming module, a vacuum heating infiltration module, a hot-press curing molding module, and a data acquisition control module. Under the conditions of natural curing at 0 MPa + 6 h + 25 °C, vacuum heating curing at –0.05 MPa + 30 min + 80 °C, and hot-press curing at 0.7 MPa + 5 min + 50 °C, a two-dimensional (2D) CFRP with excellent microstructure and properties was successfully prepared. Observing the microstructure of the prepared composite material, it can be found that the inside of the composite material was sufficiently and uniformly infiltrated, and common preparation defects such as holes and delamination were effectively controlled. Through the performance test, the bending strength of the material reached 790 MPa.

## 1. Introduction

Carbon-fiber-reinforced polymer composites (CFRPs) are widely used in the aerospace industry, weapons and equipment, transportation, electronic communications, and other fields due to their high specific strength and specific modulus, high-temperature resistance, chemical corrosion resistance, high thermal conductivity, and many other excellent properties [1,2,3]. At present, the traditional preparation methods of CFRP mainly include resin transfer molding (RTM), winding molding (WM), autoclave molding (AM), and compression molding (CM). In recent years, there were some new process preparation methods produced by improving the traditional ones, such as laser-cured automatic fiber placement molding, ultrasonic rapid curing molding, and electron beam curing molding [4,5,6].

In the existing preparation methods of CFRP, the RTM process can form complex and high-precision components, and it has the advantages of high preparation efficiency, low pollution, and strong process adaptability [7,8]. However, when the fiber volume fraction is high, there are some disadvantages such as a poor infiltration effect, high product porosity, and bad real-time controllability. The WM process is usually used to prepare tubular parts, which has the advantages of high molding accuracy and strong production reliability [9], but it has poor adaptability and high production costs. The AM process is suitable for preparing large components. It has the advantages of a stable preparation process and low porosity of the product [10], but it requires high equipment costs and large energy consumption, which cannot meet the current composite material manufacturing concept of low cost and pollution. Compared with these process methods, the CM process has the advantages of low cost, low internal stress of the product, and easy control of the process [11], but it is easy to be limited in size when preparing composite material, and the lack of resin-filled mold capabilities leads to a poor final infiltration effect. A comparison of the pros and cons of the existing traditional composite preparation process methods is shown in Table 1.

Each of the above mentioned preparation process methods has advantages and disadvantages. The promotion and application of composite materials need low cost, high efficiency, good process stability, and adaptive preparation methods as support. The existing preparation process methods have the problems of poor infiltration effect, insufficient resin-filled fiber pressure, poor process flexibility and adaptability, and inability to control the preparation process in real time when preparing composite material. In view of the above problems, this paper combines the vacuum infiltration process in the RTM and AM process with the molding process in the CM process to modularize the preparation process and control it uniformly. Finally, a vacuum infiltration hot-press forming process method is proposed innovatively, and a vacuum infiltration hot-press forming experimental system (VIHPS) is designed based on the method, allowing the high-performance CFRP to be successfully prepared. 

The VIHPS mainly includes four parts: fiber pre-forming module, vacuum heating infiltration module, hot-press curing molding module, and data acquisition control module. The cores of the VIHPS are the vacuum heating infiltration module and hot-press curing molding module. The vacuum heating infiltration module has the functions of heating and vacuuming. The fluidity of the matrix solution can be improved at high temperatures, and the vacuum negative pressure environment can enhance the infiltration effect of the solution in the fibers, effectively reducing defects such as bubbles and pores formed during the infiltration [12]. After the vacuum heating infiltration process is completed, a hard shell is formed on the outer surface of the composite material, but the interior of the material is still incompletely cured. At this time, the composite material is transferred to the hot-press curing molding module for the final heat compaction and curing. This process also effectively solves the problem of delamination failure of the material due to the easy adhesion of the composite material to the mold in the traditional compression molding process. Finally, the CFRP composite material is successfully prepared by this system. 

Through microstructure observation and a performance test of the prepared composite material, it can be found that the composite material has sufficient infiltration inside, the resin and fiber are tightly bound, and common preparation defects such as holes and delamination are effectively controlled. The bending strength of the material can reach 790 MPa. Compared with the 605 MPa of the compression molding process [13] and the 550 MPa of the RTM process [14], the maximum bending strength of CFRP prepared by VIHPS increased by 30.58% and 43.64%, respectively. Although there is still a certain gap compared with the 1096 MPa of winding molding [15], the cost of winding molding is high and the process adaptability is poor, which is only suitable for preparing tubular parts. Therefore, VIHPS is a very advantageous method. The proposal of VIHPS enriches the theory of carbon-fiber-reinforced composite material preparation methods and lays a foundation for the engineering application of CFRP.

## 2. Design of Vacuum Infiltration Hot-Press Forming Experimental System

### 2.1. Overall Design of the Experimental System

Aiming at the problems of the matrix solution having a poor infiltration effect in the fiber during the preparation of the composite material, whereby it is prone to production defects such as bubbles and pores, with the inability to monitor and feedback the production process in real time, this paper proposes VIHPS.

The basic principle of the system is that the resin is fully infiltrated in the carbon fiber preform and the surface of the material is cured in a vacuum heating environment. Then, heating and pressing are performed to eliminate the internal defects of the preform, so that the material is completely infiltrated and cured; finally, a composite material with dense tissue is obtained. 

**Figure 1:**
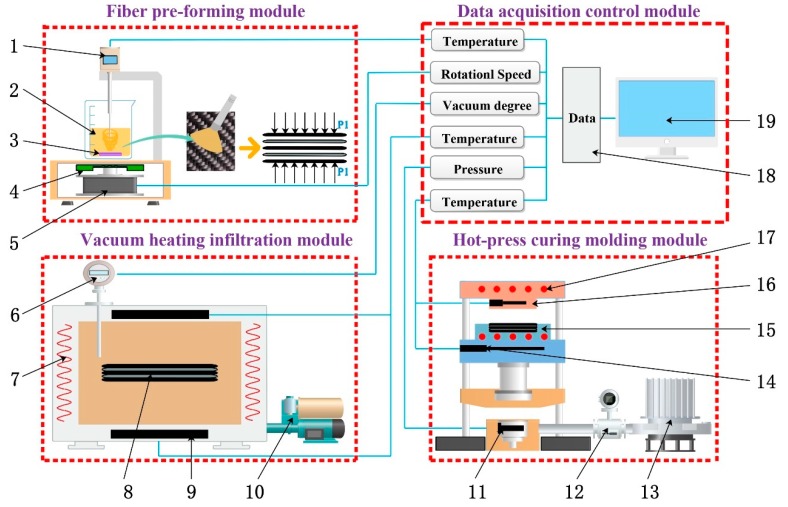
Schematic diagram of the vacuum infiltration hot-press forming experimental system (VIHPS). 1—temperature measuring instrument; 2—curing mixed solution; 3—magnetic rotor; 4—magnetic driving device; 5—motor; 6—vacuum measuring instrument; 7—heating resistance; 8—composite material; 9—temperature sensor; 10—vacuum pump; 11—pressure sensor; 12—solenoid valve; 13—hydraulic cylinder; 14—thermocouple; 15—concave die; 16—convex die; 17— heating resistance wire; 18— data acquisition instrument; 19—personal computer (PC).

The performance of composite material depends on the infiltration microstructure, and it is mainly affected by multiple process parameters such as time, temperature, vacuum, and extrusion force. Therefore, in the overall design of the system, several requirements should be fulfilled. Firstly, the two process modules of vacuum heating infiltration and hot-press curing molding should have heating functions. Secondly, multiple process parameters that affect the infiltration effect of composite materials can be collected and adjusted in real time during the experiment. Finally, in the design of the experimental system, multiple factors such as equipment cost, energy consumption, and environmental adaptability should also be considered. Based on the above ideas, our research team designed the overall structure of VIHPS, as shown in Figure 1.

### 2.2. Design of Key Parts of the Experimental System

The key parts of the VIHPS are the four modules: fiber pre-forming module, vacuum heating infiltration module, hot-press curing molding module, and data acquisition control module. The fiber pre-forming module can realize the cutting of fibers, the preparation of mixed solutions, and the optimal design of fiber layup methods, so as to finally complete the preparation process of carbon fiber preforms. The vacuum heating infiltration module can effectively reduce the air bubbles inside the fiber preform by enhancing the fluidity of the resin in the fiber under a high-temperature and negative-pressure environment, thereby achieving sufficient infiltration. The hot-press curing molding module can achieve the final complete infiltration and curing of the composite material under the action of extrusion force and higher temperature. The data acquisition control module can monitor and control the changes of key process parameter values in the process of composite material preparation in real time. The four module devices are reasonably distributed and work together to form a complete VIHPS.

#### 2.2.1. Fiber Pre-Forming Module

**Figure 2:**
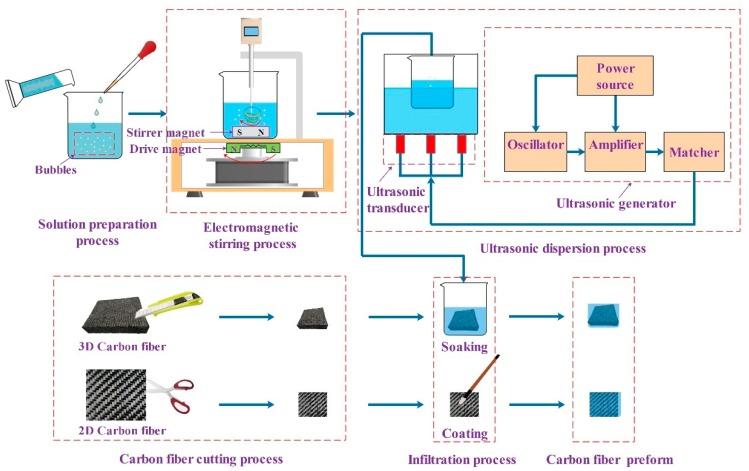
Flowchart of carbon fiber pre-forming module.

When directly using untreated carbon fibers to prepare composite material, the surface of the carbon fibers is inert [16] and cannot be well bonded with the matrix, and defects such as interfacial cracking and degumming are prone to occur, which ultimately limits the mechanical properties of the composite material [17,18]. Therefore, when preparing carbon-fiber-reinforced composite material, it is necessary to pre-treat the fibers. By uniformly dispersing the nanoparticles on the surface of the carbon fiber, the microscopic morphology of the surface of the carbon fiber is changed, and the binding ability between the fiber and the matrix is improved. The fiber pre-forming module can mainly carry out the pretreatment of carbon fiber and glass fiber, the preparation of the curing mixed solution, and the internal design and preparation of fiber preform. The specific process diagram is shown in Figure 2.

The main devices of the fiber pre-forming module include a magnetic stirrer, an ultrasonic disperser, etc. The magnetic rotor in the beaker rotates with the electromagnetic rotor in the electromagnetic stirrer, so that the solidified mixed solution inside the beaker is fully and uniformly stirred, and finally the air bubbles in the solution are effectively reduced. The stirred mixed solution is then placed in an ultrasonic disperser. Through the cavitation of ultrasonic waves, particles such as graphene, carbon nanotubes, and other nano-sized particles are dispersed in the mixed solution, thereby effectively solving the phenomenon that particles are easy to agglomerate in the solidified mixed solution [19]. At the same time, the particles are uniformly dispersed in the mixed solution, and the bubbles inside the solution can be ruptured by the action of ultrasonic waves, which further enhances the uniformity of the solution components. In the experiment, the ranges of parameters that the device can provide are as follows: speed range of the magnetic stirrer, 0–1500 rpm; working frequency of the ultrasonic disperser, 36–44 kHz.

#### 2.2.2. Vacuum Heating Infiltration Module

After the carbon fiber preform is initially prepared, the inside of the material is still in a wet state, and the solution is not completely infiltrated with the carbon fiber. The main reason is that, when the matrix solution infiltrated at room temperature, the internal temperature of the material is low, the viscosity of the matrix solution is large, and the pressure difference between the inside and outside of the capillary is small. Therefore, it is difficult for the solution to completely infiltrate the carbon fibers, so it is difficult for the solution to completely infiltrate the carbon fiber [20], and defects such as bubbles and holes are formed inside the material. When the material is loaded, these preparation defects can easily propagate into cracks, which eventually lead to failure of the material and greatly limit the performance of the material. In order to further improve the infiltration effect, according to Equation (1), Darcy’s law can indicate the infiltration depth when the matrix solution is infiltrated with carbon fibers [21].

(1) $$\frac{h^{2}}{t}=\frac{2S_{z}}{\eta (1-V_{f})}(P_{V}+P_{C}),$$

where h is the depth of infiltration of the matrix, t is the time of infiltration, $S_{Z}$ is the permeability of the reinforcement, η is the viscosity of the substrate, $V_{f}$ is the volume fraction of the fiber, $P_{V}$ is the applied vacuum pressure (pressure difference between atmosphere and the mold concave), and $P_{C}$ is the capillary pressure.

Among them, the capillary pressure can be determined by Young–Kelvin Equation (2) [22].

(2) $$P_{c}=\frac{2\gamma_{1\upsilon }\cos\theta }{r},$$

where $\gamma_{1\upsilon }$ is the surface tension of the matrix solution, θ is the wetting angle between the matrix solution and the reinforcement, and r is the capillary radius.

Substituting Equation (2) into Equation (1), we get Equation (3).

(3) $$h^{2}=\frac{2S_{Z}}{\eta (1-V_{f})}P_{V}t+\frac{4S_{Z}\gamma_{1\upsilon }\cos\theta }{\eta (1-V_{f})r}t.$$

It can be known from the above derivation that, in a certain period of time, a greater degree of vacuum in the environment in which the material is infiltrated leads to a greater depth of the matrix solution infiltrating the reinforcement, thereby improving the infiltration effect. Secondly, from the double Arrhenius viscosity model shown in Equation (4), it can be known that, when the temperature is increased, the viscosity of the matrix solution can be effectively reduced [23], thereby enhancing the fluidity of the solution and better impregnating the carbon fibers.

(4) $$\ln\eta (T,t)=\ln Z_{\infty }+\frac{E_{Z}}{RT}+K_{0}e^{-\frac{E_{T}}{RT}}t,$$

where $Z_{\infty }$ and $K_{0}$ are Arrhenius pre-factors, $E_{Z}$ is the activation energy of solution flow, $E_{T}$ is the activation energy of the curing reaction, R is the gas molar constant, T is the reaction temperature, and t is the reaction time.

**Figure 3:**
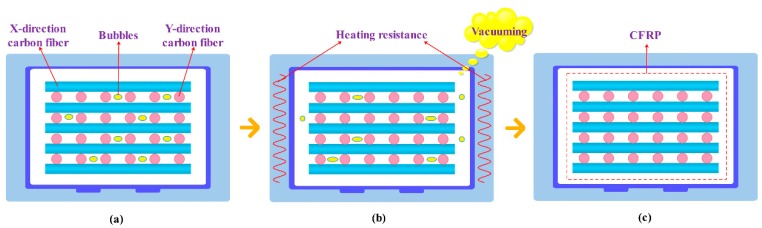
Schematic process of the vacuum heating infiltration module: (**a**) initial state; (**b**) intermediate state; (**c**) final state.

Therefore, after the fiber preform is prepared, the vacuum heating infiltration module is added to provide the degree of vacuum and temperature required for the matrix solution to infiltrate the reinforcing fiber, which can effectively improve the infiltration effect of the composite material. During the infiltration process, as shown in Figure 3a, in the initial state, the air bubbles inside the fiber preform are randomly distributed between the fibers. As shown in Figure 3b, when the vacuum heating infiltration module starts to work, a vacuum negative pressure is formed between the inside and the outside of the preform, and the internal bubbles gradually diffuse to the outer sides of the preform. At the same time, the higher temperature can improve the fluidity of the cured mixed solution in the fiber, so that the matrix solution is fully impregnated in the fiber preform. As shown in Figure 3c, defects such as bubbles and holes in the final material are effectively controlled, which reduces the possibility of cracks expanding in the material, thereby greatly improving the overall performance of the material. The vacuum heating infiltration module mainly includes an electric heating drying box, a vacuum pump, a parameter display instrument, etc. During the experiment, the module can provide a temperature range of 25–300 °C and a vacuum degree of −0.1–0 MPa.

#### 2.2.3. Hot-Press Curing Molding Module

When the composite material is infiltrated and cured in the vacuum heating infiltration module, defects such as bubbles and holes in the material are significantly improved. However, when infiltration occurs without external squeezing force, the solution preferentially flows through the gap formed by the intersection of the fiber stacks, while less of the matrix solution flows between the parallel fiber layers. When the material is finally cured, preparation defects such as delamination and warpage between the parallel fiber layers are likely to occur, which seriously affect the ultimate bending load-bearing performance of the material. By applying a certain pressing force on the outside of the material, and maintaining the material to achieve the final curing, the fiber layers can be tightly bonded, thereby greatly improving the compactness of the material. At the same time, the external pressure of the material can increase the pressure difference between the inside and outside of the capillary during the infiltration process, so that the matrix solution can effectively overcome the infiltration resistance such as viscous resistance and fiber end resistance during the infiltration. Therefore, the infiltration effect of the solution in the fiber is further enhanced and improved, and the complete infiltration of the matrix solution in the fiber reinforcement is finally achieved. 

Based on the above analysis, the designed module can provide the required pressing force. When the die applies a pressing force to the composite material, the residual strain of the solidified material is gradually distributed along the thickness direction of the material due to the thermal mismatch between the die and the material, eventually causing the material to warp and deform [24]. Therefore, the module should also have an auxiliary heating function. Based on the above work requirements, our research team designed a hot-press curing molding module.

**Figure 4:**
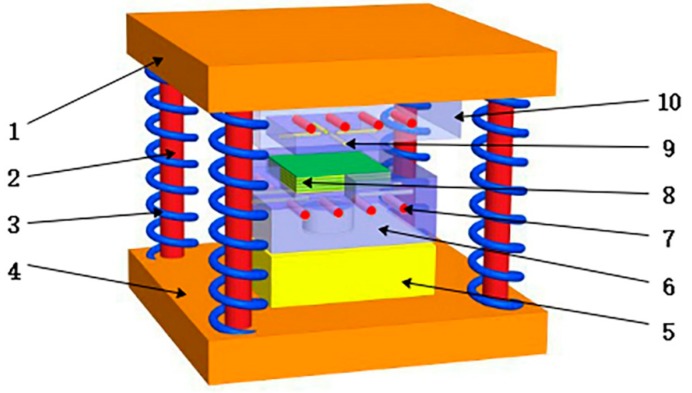
Assembly structure diagram of concave–convex die. 1—convex die fixing plate; 2—pillar; 3—spring; 4—concave die fixing plate; 5—concave die pad; 6—concave die; 7—heating resistor hole; 8—composite material; 9—thermocouple hole; 10—convex die.

The basic principle is shown in Figure 4. The convex die 10 is always fixed at the upper layer. The hydraulic cylinder provides system power to push the concave die 6 at the lower end of the device upward to squeeze the composite material. After the extrusion of the composite material is completed, the concave die is restored to its initial working position by virtue of the gravity of the concave die and the restoring force of the four springs on the periphery of the device. There are heating holes 7 and thermocouple detection holes 9 on both sides of the concave–convex mold to realize the heating, thermal insulation, and temperature monitoring of the mold. The overall design and assembly structure diagram of the concave–convex mold is shown in Figure 4.

There are several factors that need to be considered when determining the parameters of a hot-press curing module, for example, the size range of the prepared samples, the design of the concave-convex mold and the choice of mold materials, the selection of hydraulic presses, the placement of molds, the layout of heating resistors and thermocouples, etc. The strength check in the design of the concave–convex mold and the calculation of the heating power required by the device are the keys to the entire hot-press curing molding module. For the strength check of the concave–convex die, the concave die design may not be checked based on the empirical formula, but the convex die design still needs to be checked for performance. The verification of the convex die mainly involves two aspects: compressive stress check and bending stress check. The specific calculation process is as follows:

(1) Compressive stress check of convex die

The minimum cross-sectional area of a rectangular punch should satisfy Equation (5).

(5) $$A_{\min}\geq \frac{p_{0}}{\left[\sigma_{c}\right]},$$

where $p_{0}$ is the mold pressure, and $\left[\sigma_{c}\right]$ is the allowable compressive stress of the material. The material of the convex die is 45 steel, so $[\sigma_{c}]=190\;\mathrm{MPa}$. The selected hydraulic machine is a Y32t jack, which can provide a maximum pressure of 320 kN, so $p_{0}=3.2\times 10^{5}N$, and $A_{\min}=1684.21\;mm^{2}$. The design takes the convex die side length of 100 mm, so the design passes the compressive stress check.

(2) Bending stress check of convex die

The maximum free length of a rectangular punch should satisfy Equation (6).

(6) $$L_{\max}\leq 420\sqrt{\frac{J_{}}{P_{0}}},$$

where J is the moment of inertia of the smallest cross-sectional area from the convex die to the mandrel. Since the cross-sectional area is rectangular, J can be calculated by $J=a^{4}/12$, where a = 50 mm; thus, $J=520,833.33\;{\mathrm{mm}}^{4},L_{\max}=535.83\;\mathrm{mm}$. The device design value is 119.6 mm, which is less than 535.83 mm, so it passes the bending stress check.

The total power required for the electric heating device of the mold can be calculated by Equation (7).

(7) $$W=\frac{Vc\gamma T_{2}-T_{1}}{3.588\times 10^{3}}+0.31A_{b},$$

where V is the mold volume, γ is the density of the mold material, c is the specific heat capacity of the steel, $T_{2}$ is the ideal preheat temperature, $T_{1}$ is the room temperature, $A_{b}$ is the total exposed surface area of the mold, 0.31 is the heat loss coefficient per unit exposed surface area, and $3.588\times 10^{3}$ is the power conversion factor. 

Bringing known parameters γ=7.8 g/cm3,c=0.460 J/(g·C°) into Equation (7), it can be simplified to Equation (8).

(8) $$W=0.001V(T_{2}-T_{1})+0.31A_{b},$$

where V=10644 cm3,T1=25 C°,T2=200 C°,Ab=2812 cm2; thus, the required heating power of the device calculated according to this equation is W=2734.42w. During the experiment, the pressure value provided by the hot-press curing molding module ranges from 0 to 32 MPa, and the heating temperature ranges from 0 to 200 °C.

#### 2.2.4. Data Acquisition Control Module

In the process of preparing composite material, the process parameters such as temperature, time, pressure, and vacuum play a decisive role in the final performance of the material, based on the Arrhenius Equation (9).

(9) $$k=Ae^{-E_{a}/RT},$$

where k is the reaction rate constant, A is the Arrhenius index factor, $E_{a}$ is the reaction activation energy, R is the gas molar constant, and T is the curing infiltration temperature.

It can be known from Equation (9) that, when the curing infiltration temperature increases, the infiltration and curing speed inside the material obviously increase, and the time required to reach the required degree of curing decreases. Therefore, when the temperature is too high, the matrix solution rapidly solidifies and does not sufficiently infiltrate the fiber preform. Secondly, higher temperature reduces the stiffness and failure strength of the material [25]. When the temperature is low, it can be known from Equation (4) that the matrix has a large viscosity, the solution has poor fluidity, and the time required for the infiltration reaction process is too long, which is not conducive to the infiltration process. 

During the preparation process, the external pressing force should also be selected within a suitable parameter range. When the external pressure is low, the inside of the material cannot be tightly bonded, and defects such as delamination and warpage are eventually prone to occur. When the external pressing is too large, it is easy to crush a part of the solidified material, eventually causing the failure of the material. It can be known from Equation (1) that the degree of vacuum determines the capillary pressure difference during the internal infiltration of the material, which in turn affects the elimination of defects such as bubbles and holes in the material and the improvement of the infiltration effect.

**Figure 5:**
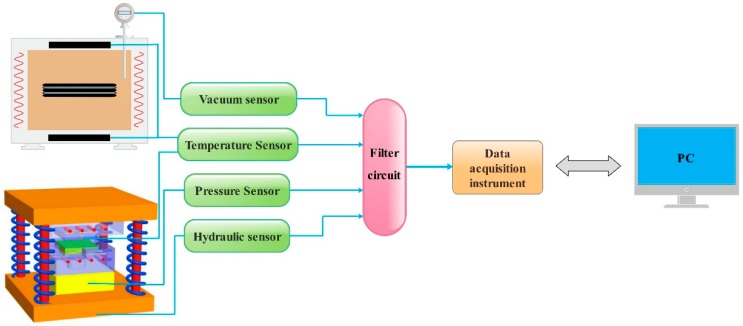
Schematic diagram of the structure and principle of the VIHPS parameter acquisition and control system.

Therefore, in the preparation process, key process parameters such as temperature, time, material, pressure, and vacuum degree need to be collected and controlled in real time, so as to ensure that the composite material is prepared and formed under reasonable process parameters. Based on the above analysis, our research team designed a data acquisition control module, and its acquisition control principle is shown in Figure 5.

The data acquisition control module mainly includes a pressure sensor controller, a temperature acquisition controller, a vacuum degree digital display instrument, and a 10-channel data acquisition instrument. When preparing composite materials, a series of sensors are used to collect parameters such as temperature, vacuum, pressure, and hydraulic pressure values and convert them in real time through a filter circuit. Then, the relevant parameters are presented by the data acquisition instrument and finally transmitted to the computer, and the changes of the main process parameters in the preparation process are monitored by the computer in real time. When the parameters are abnormal, the fault parameters can be adjusted in time to provide a guarantee for the preparation of composite materials with ideal microstructure and properties.

**Figure 6:**
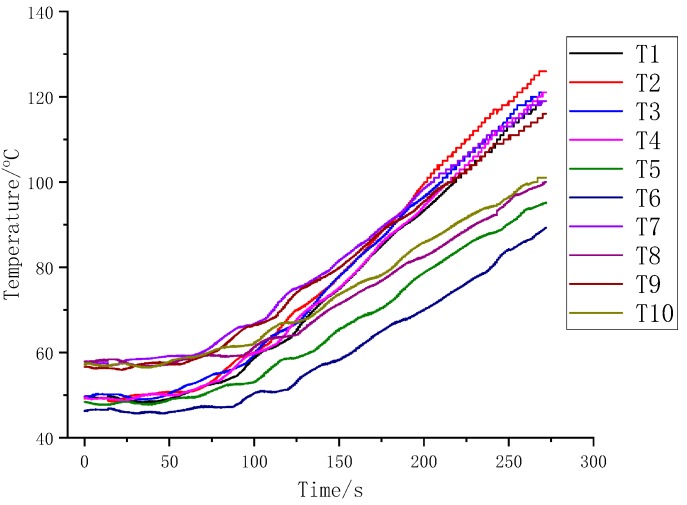
Typical temperature acquisition curve during module work.

The variation curve of typical temperature parameters collected during the preparation process is shown in Figure 6. In order to monitor the temperature of the mold and make it evenly heated, 10 thermocouple detection holes at different positions and depths are set inside the upper and lower surfaces of the concave–convex mold to collect the temperature change of the mold in real time. In this way, the temperature of the mold is consistent with the surface temperature of the material, and the adverse effects of thermal mismatch of the material during the preparation process are eliminated. From the data collected in Figure 6, it can be known that the mold temperature can be increased from 50 °C to 130 °C in about 250 s, which ensures that the test process is stable, reliable, and fast.

### 2.3. Physical Construction of Vacuum Infiltration Hot-Press Forming Experimental System

**Figure 7:**
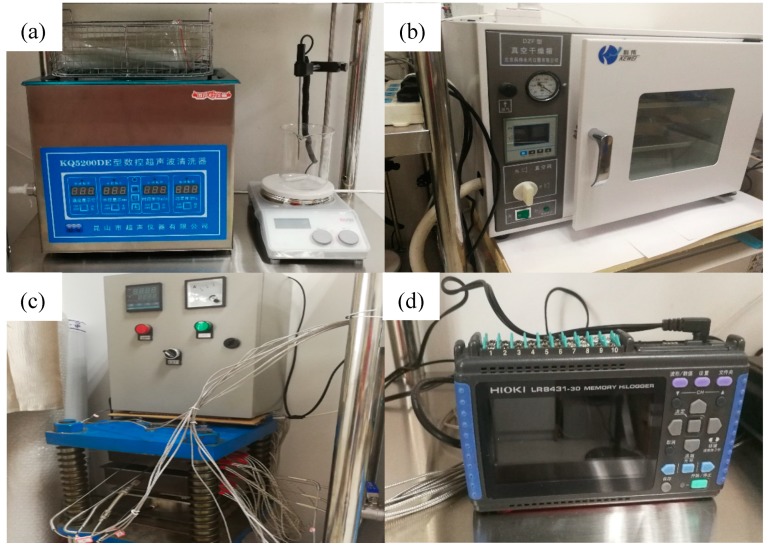
Main instrument and equipment of the vacuum infiltration hot-press forming experimental system: (**a**) ultrasonic cleaning and magnetic stirrer; (**b**) vacuum heating infiltration box; (**c**) hot-press forming and temperature control device; (**d**) data acquisition instrument.

The above sections analyzed the design ideas and structural principles of the vacuum infiltration hot-press forming experimental system in detail. Under the control of reasonable process parameters, the four modules of fiber pre-forming, vacuum heating infiltration, hot-press curing molding, and data acquisition control cooperate with each other and work together to prepare a composite material with excellent comprehensive performance. Based on the design analysis of the principle and composition of the system, our research team realized the physical construction of the vacuum infiltration hot-press experimental system. Figure 7a shows the main equipment of the fiber pretreatment module, i.e., the ultrasonic cleaner and magnetic stirrer, which can realize the configuration of the solidified mixed solution. Figure 7b shows the vacuum heating infiltration box, which can realize the infiltration of the material under the vacuum heating environment. Figure 7c shows the hot-press curing mold and temperature control device, which can realize the heating and extrusion of composite material. Figure 7d shows the main device of the data acquisition control module, i.e., a 10-channel data acquisition instrument. It can collect multiple process parameters, such as temperature, pressure, and vacuum, in real time during the preparation process, so as to provide real-time feedback on the preparation process.

## 3. Composite Material Preparation Experiment

### 3.1. Experimental Materials

The matrix used in the experiment was E-44 epoxy resin produced by Xi’an Resin Factory in Xi’an, China. The reinforcement was 12K unidirectional T700 carbon fiber produced by Toray in Tokyo, Japan, and its related physical and mechanical properties are shown in Table 2. The curing agent used was the 593 curing agent produced by Jiangsu Sanmu Group Co., Ltd in Yixing, China.

### 3.2. Experimental Process of Two-Dimensional (2D)-CFRP Preparation

The designed vacuum infiltration hot-pressing experimental system was used to prepare CFRP composite materials, and the feasibility of the experimental system was verified. The preparation process mainly included cutting and pretreatment of carbon fiber, preparation and pre-infiltration of curing mixed solution, natural curing of a composite material, vacuum infiltration curing, and hot-press curing molding.

**Figure 8:**
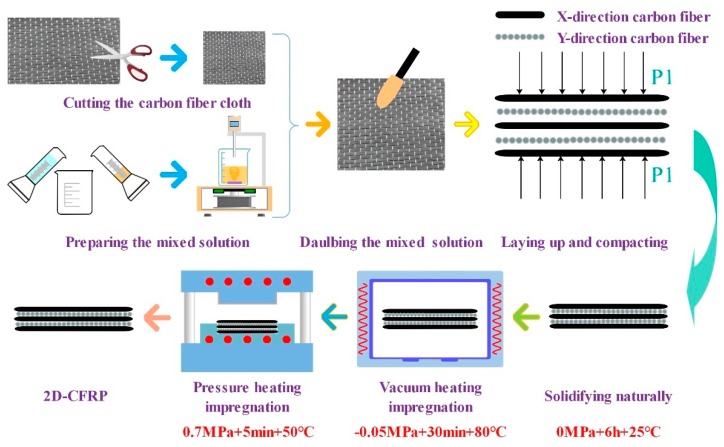
Schematic diagram of the process of preparing 2D-CFRP by the VIHPS.

The specific process incorporated five steps. Firstly, the T700 carbon fiber cloth was cut into (60 mm × 60 mm) square pieces, the epoxy resin and the curing agent were mixed at a mass ratio of 4:1, and the mixture was placed in an electromagnetic stirrer to stir it uniformly. Secondly, the prepared curing mixed solution was applied evenly on both sides of the cut carbon fiber cloth, and then stacked in order; the angle between adjacent fiber layers was 90°, and certain pressure was applied to pre-compact the layers. Thirdly, the prepared carbon fiber laminate was cured at 25 °C for 6 h, and then transferred to the vacuum heating infiltration module, where the degree of vacuum was −0.05 MPa, the curing time was 30 min, and the temperature was 80 °C. Fourthly, the carbon fiber laminate was removed and placed it into the hot-press curing molding module, where the extrusion force was 0.7 MPa, the holding time was 5 min, and the temperature was 50 °C. Lastly, the heating and pressing process was stopped, and, once the mold returned to room temperature, the prepared 2D-CFRP composite material was removed. The main process parameters for preparing the 2D-CFRP composite material by VIHPS are shown in Table 3, and a schematic diagram of the process flow is shown in Figure 8.

### 3.3. Testing and Characterization Methods

The 2D-CFRP composite material was successfully prepared by the vacuum infiltration hot-press forming experimental system. A JEOLJSM-6390A scanning electron microscope produced by Japan Electronics Co., Ltd in Tokyo, Japan was used to observe the infiltrated microstructure and bending fracture morphology of the composite. The DNS100 electronic universal testing machine made by Changchun Institute of Mechanical Science in Changchun, China was used to test the three-point bending strength of the composite. The specific test method was in accordance with the “Testing Method for Flexural Performance of Fiber-Reinforced Plastics” (GB/T1449-2005), in which the size of the sample used was 50 mm × 15 mm × 2 mm, the span was 40 mm, the radius of the loading head was 5 mm, and the test loading speed was 10 mm/min.

### 3.4. Experimental Results

**Figure 9:**
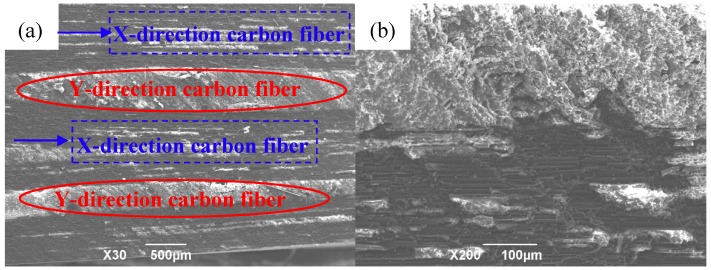
Infiltration microstructure of 2D-CFRP composite: (**a**) Infiltration microstructure of 2D-CFRP (×30); (**b**) infiltration microstructure of 2D-CFRP (×200).

As shown in Figure 9a, the infiltrated microstructure of the prepared composite material was observed, in which the white portion was the matrix and the black portion was the carbon fiber. The adjacent layers of the fibers were distributed at 90°, the infiltration effect of the composite material was good, and the layers were tightly bonded. The matrix was evenly distributed between the carbon fibers, and the two were tightly combined without obvious manufacturing defects such as holes and pores. When the magnification was further increased to 200×, as shown in Figure 9b, the infiltration microstructure of the composite material was still ideal. The matrix and carbon fiber were tightly bonded without microcracks due to poor compatibility. When the composite material was loaded, the carbon fiber could play a reinforcing role, and the resin matrix could transfer the stress. Therefore, the maximum bending performance of the composite material was greatly improved, and the bending strength of the composite material was 790 MPa.

**Figure 10:**
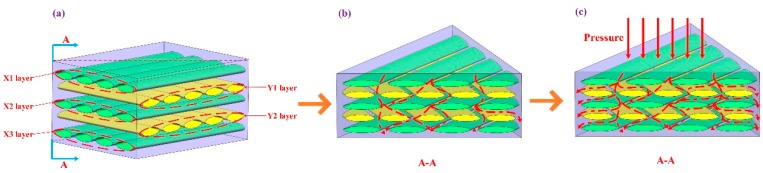
Carbon fiber distribution model and infiltration mechanism: (**a**) carbon fiber structure distribution model; (**b**) initial infiltration of material in no pressure; (**c**) full infiltration of material in pressure.

As shown in Figure 10a, the 2D-CFRP was prepared by turning the angle between adjacent layers of unidirectional T700 carbon fiber at 90°. When no external load was applied in the initial stage of composite material preparation, the solution preferentially flowed downward in the direction of less resistance, i.e., the channel was formed by the intersection of the X1 layer and the Y1 layer, and the X2 and the Y2 layer. There was less solution infiltrated between the X1 layer and the Y1 layer, as shown in Figure 10b. As the solidified mixed solution gradually infiltrated the fibers, the channels with less resistance were gradually filled. At this time, the external pressing force applied to the material could effectively guide the solution to flow between the fiber layers and infiltrate each carbon fiber in the fiber bundle. As shown in Figure 10c, sufficient infiltration of the composite material was finally achieved.

**Figure 11:**
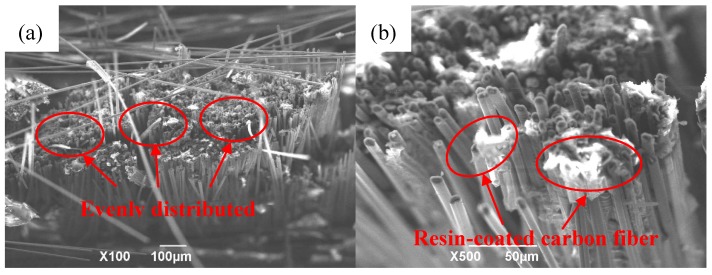
Bending fracture morphology of 2D-CFRP composites: (**a**) bending fracture of 2D-CFRP (×100); (**b**) bending fracture of 2D-CFRP (×500).

The bending fracture morphology of the prepared CFRP composite was observed, as shown in Figure 11a. The breaking and pulling out of fibers in the fracture of the composite material indicated that the resin was fully infiltrated between the carbon fibers and the two were tightly combined. When the magnification was further increased, as shown in Figure 11b, it can be found that the resin was coated inside and between the carbon fiber bundles, while also reflecting that the material’s infiltration effect was indeed greatly improved. Furthermore, it can be seen that the vacuum infiltration hot-press forming process method could be applied to the preparation of CFRP. The proposal of VIHPS enriches the preparation process system of CFRP and provides experience for the promotion and engineering application of CFRP composite materials. 

## 4. Conclusions

(1) In this paper, a vacuum infiltration hot-press process method was proposed innovatively. Based on this method, a vacuum infiltration hot-press process experimental system was designed and developed. The system mainly includes a fiber pre-forming module, a vacuum heating infiltration module, a hot-press curing molding module, and a data acquisition control module. The modular division of the preparation process and unified data collection monitoring greatly improve the stability and controllability of the preparation process. In addition, the VIHPS has the advantages of low production cost and excellent product performance, which lays a foundation for the preparation and market application of CFRP composite materials.
(2) Curing time, temperature, pressure, and vacuum degree are important process parameters for preparing CFRP by VIHPS. These key parameters directly affect the infiltration effect of the composite material, and finally affect the macroscopic properties of the material. Therefore, these important parameters need to be strictly controlled during the preparation process. The experimental research showed that, when the conditions of natural curing at 0 MPa + 6 h + 25 °C, vacuum heating curing at −0.05 MPa + 30 min + 80 °C, and hot-press curing at 0 MPa + 5 min + 50 °C were applied, the performance of the prepared composite material was the best, and the bending strength of the composite material reached 790 MPa.
(3) By observing and analyzing the CFRP infiltration structure and bending fracture morphology prepared under reasonable process parameters, it can be found that the infiltration structure inside the material was ideal, and the resin was evenly distributed between the carbon-fiber bundles without obvious holes, delamination, warping, and other preparation defects. The resin matrix was tightly combined with the carbon fiber. From the bending fracture morphology of the composite material, it can also be found that the carbon fiber was in a fractured and pulled out state, the resin was evenly distributed in the carbon fiber, and the internal microstructure of the material was ideal, thereby confirming the feasibility of the vacuum impregnation hot-press molding process test system.

## Figures and Tables

**Table 1:** Pros and cons of traditional carbon-fiber-reinforced polymer composite (CFRP) preparation process methods.

Process Methods	Resin Transfer Molding	Winding Molding	Autoclave Molding	Compression Molding
Pros	◆ Efficiency ◆ Little pollution ◆ Adaptability	◆ Tubular ◆ Productivity ◆ Reliability	◆ Large parts ◆ Low porosity ◆Stability	◆ Cost ◆ Low internal stress ◆ Controllability
Cons	◆ High porosity ◆ Controllability	◆ Cost ◆ Adaptability	◆ Energy consumption ◆ Cost	◆ Infiltration effect ◆ Preparation efficiency

**Table 2:** Physical and mechanical properties of 12K unidirectional T700 carbon fiber.

Fiber Type	Tensile Strength (MPa)	Young’s Modulus (GPa)	Density (g/cm^3^)	Elongation (%)	Monofilament Diameter (10^−6^ m)
T700	4900	230	1.80	2.0	7

**Table 3:** Process parameters for preparing two-dimensional (2D)-CFRP composite materials by the VIHPS.

Curing Mixed Ratio/ Mass Ratio	Natural Curing Time (h)	Vacuum Heating Curing Temperature (°C)	Vacuum Heating Curing Time (min)	Pressure Heating Curing Temperature (°C)	Pressure Heating Curing Pressure (MPa)	Pressure Heating Curing Time (min)
4:1	6	80	30	50	0.7	5
